# Outpatient Total Extraperitoneal (TEP) for inguinal hernia repair following federal German outpatient operation procedure reform: a preliminary single-center descriptive study of same-day discharge, unplanned admission, and patient-reported outcomes

**DOI:** 10.1186/s12893-026-04161-y

**Published:** 2026-08-27

**Authors:** Julia Michel, Krzysztof Nowakowski, Fabian Nimczewski, Jens Hoeppner, Zsolt Madarasz

**Affiliations:** Department of Surgery, Medical School, Bielefeld University, University Medical Center OWL, Campus Hospital Lippe, Röntgenstr. 18, Detmold, 32765 Germany

## Abstract

**Background:**

Outpatient hernia repair is becoming increasingly important, particularly with the adoption of minimally invasive techniques such as total extraperitoneal repair (TEP). Following the 2022 reform of the federal German outpatient operation procedure (AOP) catalog, our institution implemented a planned outpatient TEP pathway for selected patients with unilateral inguinal hernia.

**Methods:**

This preliminary retrospective single-center study included a core cohort of 108 patients who were successfully discharged on the day of surgery after planned outpatient TEP repair between January 2022 and May 2025. A separate analysis included all 153 patients initially scheduled for outpatient TEP repair, of whom 45 required unplanned inpatient admission. Of the 108 patients in the core cohort, 65 (60.2%) completed structured telephone follow-up interviews. Patient-reported outcomes were evaluated exclusively in this subgroup.

**Results:**

Among all 153 patients initially scheduled for outpatient TEP repair, 108 (70.6%) were successfully discharged on the day of surgery, whereas 45 (29.4%) required unplanned inpatient admission. Among the 108 same-day discharged patients, seven perioperative complications (four intraoperative and three postoperative) were recorded, with no life-threatening bleeding, emergency reoperation, or ICU admission. Patient-reported outcomes were available for 65 of these 108 patients (60.2%). Among respondents, 95.4% stated that they would choose outpatient surgery again, and overall satisfaction was 4.6 ± 0.8 on a 5-point Likert scale (63 valid responses).

**Conclusion:**

This preliminary single-center experience provides descriptive information on the early implementation of an outpatient TEP pathway under the German AOP framework. Although patients who successfully completed same-day discharge and responded to follow-up reported high satisfaction and perceived safety, the 29.4% unplanned inpatient admission rate, exclusion of admitted patients from the patient-reported outcome analysis, and 60.2% response rate substantially limit conclusions regarding the overall safety, acceptability, and successful implementation of the programme. Prospective multicenter studies with predefined admission criteria and systematic follow-up of all patients are required.

**Supplementary Information:**

The online version contains supplementary material available at 10.1186/s12893-026-04161-y.

## Introduction

Outpatient surgical procedures are gaining increasing attention in the context of healthcare policy reforms and the implementation of the outpatient operation procedure (AOP) directive in Germany. As part of the AOP strategy, hospitals are increasingly required to perform certain procedures—such as elective inguinal hernia repair—on an outpatient rather than inpatient basis. This shift is driven not only by regulatory requirements set forth by the Federal Joint Committee and Section 115b of the German Social Code but also by financial incentives [1].

The reimbursement for an outpatient TEP (total extraperitoneal) procedure typically ranges from €500 to €700, depending on the insurance provider and federal state. In contrast, inpatient reimbursement under the DRG system averages between €2,000 and €2,500. Although the inpatient rate is nominally higher, the actual financial return is often diminished by hospital infrastructure and personnel costs. In many cases, outpatient procedures—despite their lower nominal reimbursement— are often more cost-effective due to significantly reduced process-related expenses [2, 3].

International studies further demonstrate that outpatient interventions can achieve substantial cost savings without compromising quality when compared to inpatient settings [4, 5]. Against this background, outpatient TEP may represent a clinically appropriate and potentially cost-effective option in selected patients.

Initially, outpatient TEP procedures were historically viewed with skepticism in surgical discourse, particularly due to concerns over potential bleeding in the preperitoneal space—complications that may be difficult to detect or manage in a non-clinical environment [6]. International guidelines emphasize that patients with coagulation disorders or ongoing anticoagulation represent a higher-risk group for perioperative complications and therefore require careful patient selection when considering outpatient inguinal hernia repair [7]. To mitigate these risks, our institution established strict inclusion criteria for outpatient TEP, including ASA physical status I–III and no relevant anticoagulation therapy, and implemented a structured follow-up protocol.

The aim of this preliminary single-center study was to descriptively characterize the early implementation of an outpatient TEP pathway following the German AOP reform, including same-day discharge outcomes, patterns of unplanned inpatient admission, and patient-reported outcomes among patients who successfully completed outpatient treatment.

## Materials and methods

### Study design and patient selection

This retrospective single-center study evaluated a core cohort of 108 patients who were successfully discharged on the day of surgery after planned outpatient unilateral total extraperitoneal (TEP) inguinal hernia repair between January 2022 and May 2025. In addition, all 153 patients initially scheduled for outpatient TEP during the study period were included in a separate analysis of unplanned inpatient admission. Inclusion criteria comprised unilateral inguinal hernia, American Society of Anesthesiologists (ASA) physical status classification I–III, absence of relevant anticoagulation therapy or coagulation disorder, and a stable social environment allowing for safe discharge on the day of surgery.

German outpatient surgery framework and institutional outpatient TEP protocol.

In Germany, hospital-based outpatient surgery is regulated under Section 115b of the German Social Code (SGB V) and implemented through the German outpatient operation procedure catalogue (AOP catalogue) [1]. This framework defines procedures that may be performed as outpatient or day-case procedures but does not replace individual clinical risk assessment. Therefore, implementation of outpatient TEP at our institution was based on a structured local protocol combining the national outpatient surgery framework with procedure-specific clinical selection criteria.

According to our institutional protocol, patients were considered eligible for outpatient TEP if they had a unilateral inguinal hernia, ASA physical status I–III, no relevant anticoagulation therapy or coagulation disorder, and a stable social environment allowing safe same-day discharge. Patients were informed preoperatively about the outpatient setting, warning symptoms, and the planned postoperative follow-up. Discharge on the day of surgery required stable vital signs, adequate pain control, ability to mobilize, absence of clinically relevant bleeding suspicion, and availability of postoperative support. All patients were scheduled for clinical follow-up and dressing change on the first postoperative day.

The relationship between the German outpatient surgery framework and the institutional outpatient TEP protocol is summarized in Table 1.

**Table 1:** Relationship between the German outpatient surgery framework and the institutional outpatient TEP protocol

Domain	German outpatient surgery framework	Institutional outpatient TEP protocol
Regulatory basis	AOP framework under Section 115b SGB V	Local implementation at University Medical Center OWL – Campus Hospital Lippe
Procedure eligibility	Selected procedures may be performed as outpatient or day-case procedures if clinically appropriate	Planned outpatient unilateral TEP repair
Patient selection	Individual clinical assessment remains required	ASA I–III, unilateral inguinal hernia, no relevant anticoagulation therapy or coagulation disorder, stable social environment
Perioperative management	Outpatient treatment possible if same-day discharge is clinically appropriate	Standardized TEP under general anesthesia, PACU monitoring, predefined discharge criteria
Safety escalation	Inpatient admission remains possible if medically necessary	Low threshold for unplanned inpatient monitoring in case of bleeding suspicion, pain, cardiovascular events, or social/organizational factors
Follow-up	No procedure-specific follow-up pathway defined by the AOP catalogue	Routine clinical review and dressing change on postoperative day 1

### Surgical procedure and perioperative management

All procedures were performed using a standardized TEP technique under general anesthesia. Postoperatively, patients were monitored in the post-anesthesia care unit (PACU). Same-day discharge was permitted only if predefined clinical discharge criteria were fulfilled, including stable vital signs, adequate pain control, absence of clinically relevant bleeding suspicion, ability to mobilize, and availability of postoperative support. As part of the institutional outpatient pathway, all patients were routinely scheduled for clinical follow-up and dressing change on the first postoperative day, generally performed by the operating surgeon. Actual attendance at this review was not systematically recorded in the study dataset. In cases of intraoperative concerns, postoperative symptoms, cardiovascular events, social factors, or suspected intraoperative bleeding requiring observation, patients were admitted with a low threshold for precautionary monitoring. For the purposes of this study, “diffuse minor bleeding” referred to intraoperative diffuse oozing without an identifiable bleeding vessel and without hemodynamic instability or the need for reoperation. In contrast, “suspected intraoperative bleeding requiring observation” referred to an intraoperative concern that prompted precautionary inpatient monitoring, irrespective of whether a clinically significant hemorrhage was subsequently confirmed. Estimated blood loss was not systematically quantified in the retrospective records; therefore, no numerical blood-loss threshold could be applied.

### Data collection and follow-up

Retrospective follow-up was conducted using standardized telephone interviews based on a structured questionnaire specifically developed for this study to assess patient-reported outcomes (see Supplementary File 1). Before each telephone interview, patients were informed about the purpose of the survey and provided verbal informed consent to participate. The interviews were conducted during a single follow-up period in April and May 2025 by two authors (J.M. and Z.M.) and the departmental secretary, all affiliated with the treating department. Consequently, the interval between surgery and interview varied substantially across patients treated between January 2022 and May 2025. The questionnaire assessed overall satisfaction, perceived safety after discharge, recovery time, and willingness to undergo outpatient surgery again. In addition, documented Numeric Rating Scale (NRS) pain scores from the PACU as well as administered analgesics were extracted from anesthesia records.

### Outcome measures and statistical analysis

Primary patient-reported endpoints were satisfaction and perceived safety among respondents who had successfully completed same-day discharge. Secondary endpoints included postoperative pain, perioperative complications, unplanned conversion to inpatient care, and recovery time. Data were analyzed descriptively and are reported as absolute numbers, percentages, and means ± standard deviation, as appropriate. Due to the descriptive nature of this retrospective quality analysis, no inferential statistical testing was performed.

### Conversion analysis

The core study cohort comprised the 108 patients successfully discharged on the day of surgery; patient-reported outcomes were available for 65 of these patients. In contrast, the conversion analysis included all 153 planned outpatient unilateral TEP procedures performed during the study period to descriptively characterize the frequency and reasons for unplanned inpatient admission. Reasons for inpatient admission were categorized into predefined clinical groups and analyzed descriptively. This analysis was intended to provide additional information on the early implementation of the outpatient pathway beyond the patient-reported outcomes of the core cohort.

### Ethics approval

The study was reviewed by the Ethics Committee of the Medical Association of Westphalia-Lippe and Bielefeld University (reference number: 2025-519-f-S). Due to the retrospective nature of this anonymized quality assurance analysis using routine clinical data, written informed consent was not required according to local regulations.

## Results

### Patients and procedures

**Fig. 1:**
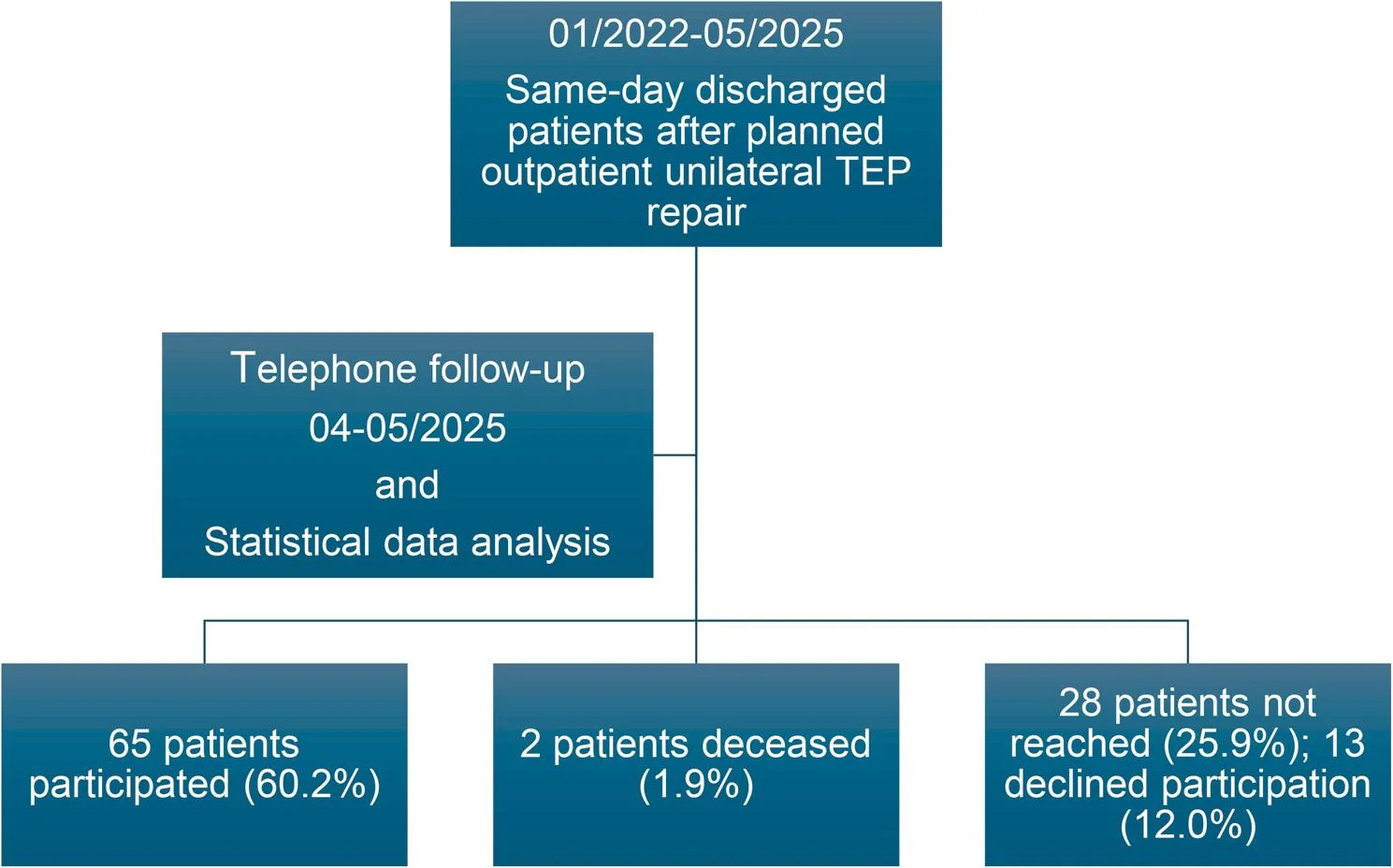
Patient inclusion and follow-up

A total of 153 patients were scheduled for outpatient unilateral TEP repair during the study period. Of these, 108 patients were successfully discharged on the day of surgery and constituted the core study cohort, whereas 45 patients required unplanned inpatient admission. Figure 1 illustrates the core study cohort, telephone follow-up, and reasons for non-participation.

Of 108 patients, 65 (60.2%) completed structured telephone follow-up and were included in the patient-reported outcomes analysis; 28 patients (25.9%) could not be reached, 13 (12.0%) declined participation, and 2 (1.9%) had died before follow-up. One patient died from advanced renal cell carcinoma two months after TEP surgery. The second patient died approximately one month before the telephone survey, more than one year after surgery performed in early 2024; the cause of death was unavailable, but no procedure-related complication had been documented.

The cohort was predominantly male, with a mean age of 55.9 ± 12.3 years and a mean body mass index of 25.2 ± 3.8 kg/m². The majority of patients were classified as ASA II, while only 5.6% had ASA III (Table 2), reflecting a predominantly low-risk cohort selected for outpatient care. Lateral hernias were the most common primary inguinal hernia type, occurring in 66 of 108 patients (61.1%). Concomitant femoral and obturator hernias were recorded separately. Mean operative time was 39.7 ± 11.1 min, and the average length of stay was 6 h 47 min ± 49 min. Baseline patient characteristics and perioperative parameters are summarized in Table 2.

**Table 2:** Baseline characteristics of patients undergoing outpatient TEP procedure (*n* = 108)

Variable	Value (Mean ± SD or *n* [%])
Demographics	
Male	102
Female	6
Age (years)	55.9 ± 12.3
Body Mass Index (kg/m²)	25.2 ± 3.8
ASA classification	
ASA I	39 (36.1%)
ASA II	63 (58.3%)
ASA III	6 (5.6%)
**Hernia morphology**	
→Primary inguinal hernia type	
Lateral	66 (61.1%)
Medial	18 (16.7%)
Combined	24 (22.2%)
→Additional concomitant hernias	
Femoral	2 (1.9%)
Obturator	1 (0.9%)
**Surgical parameters**	
Operative time (minutes)	39.7 ± 11.1
Length of stay (hours: minutes)	6:47 ± 0:49

*Abbreviations: *ASA* American Society of Anesthesiologists

Primary inguinal hernia morphology was classified as lateral, medial, or combined. Cases with both medial and lateral components were classified as combined hernias. Concomitant femoral and obturator hernias were recorded separately and were not included in the primary inguinal hernia classification

### Operative results

Operative outcomes and perioperative complications are summarized in Table 3. Four intraoperative bleeding-related events were documented in the core cohort (4/108, 3.7%). Three consisted of diffuse minor bleeding without an identifiable bleeding vessel; drains were placed in two of these patients as a precautionary measure. In one additional patient, bleeding from the epigastric vessels was controlled by clipping. None of these patients developed a subsequent bleeding-related complication, required reoperation, or required intensive care treatment. Postoperatively, one patient (1/108, 0.9%) developed a hematoma that was managed conservatively, and two early recurrences (2/108, 1.9%) were observed and later treated with open mesh repair. No life-threatening bleeding, emergency reoperation, or ICU admission occurred.

**Table 3:** Operative results and postoperative complications

Variable	Value (*n*)
Intraoperative complications	
⤍Diffuse minor bleeding without identifiable source	3
⤍Bleeding from epigastric vessels controlled by clipping	1
Postoperative complications	
⤍Hematoma	1
⤍Early recurrence	2

The three cases of diffuse minor bleeding involved intraoperative oozing without an identifiable bleeding vessel; drains were placed in two patients as a precautionary measure

### Patient reported outcomes

Postoperative pain scores in the PACU were available for 54 of 108 patients (50.0%) and were low overall (mean NRS 1.3 ± 2.2).

Preoperative counseling was rated positively by the majority of respondents: 23 patients (35.4%) rated it as “very good,” 33 (50.8%) as “good,” and 8 (12.3%) as “satisfactory.” No ratings of “poor” or “very poor” were reported. One patient (1.5%) could not recall the preoperative information.

**Fig. 2:**
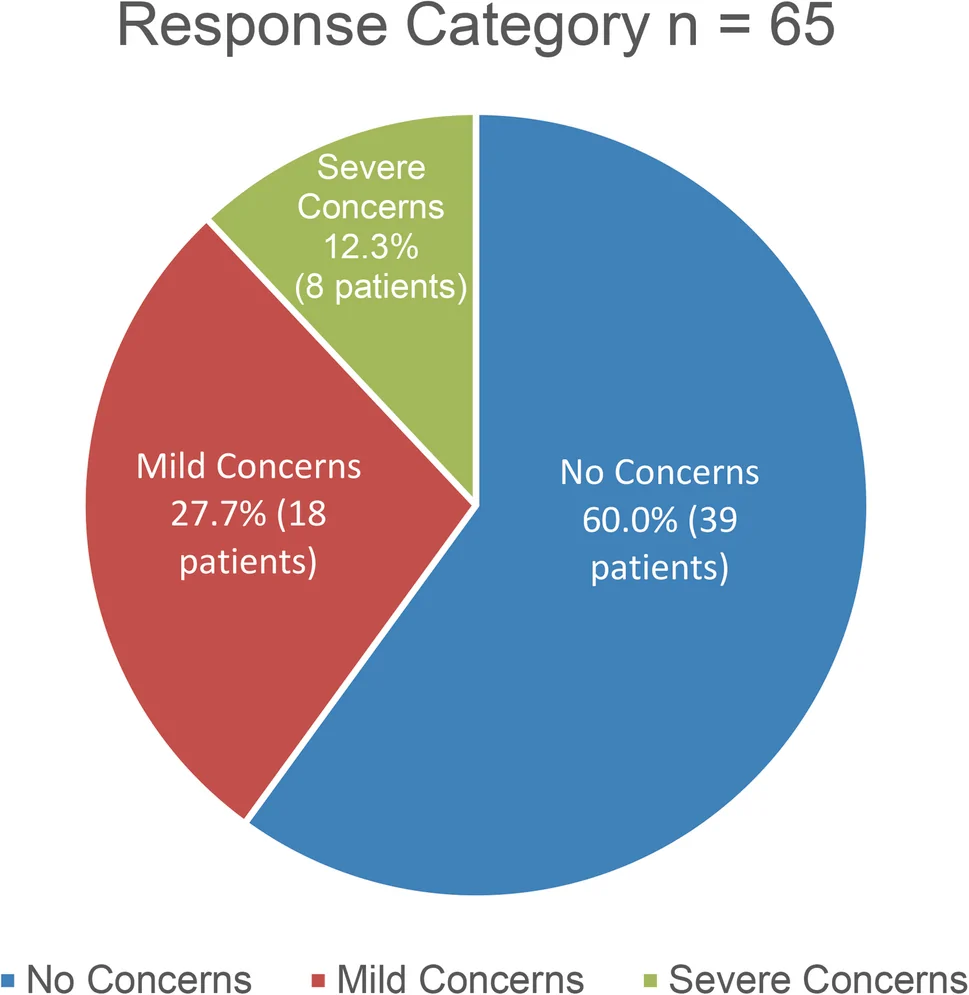
Preoperative concerns among patients undergoing outpatient TEP surgery (*n* = 65)

Most patients reported no significant concerns prior to surgery: 60.0% (39 patients) indicated they had no concerns before the procedure, 27.7% (18 patients) reported mild concerns, and 12.3% (8 patients) expressed strong concerns. Overall, significant preoperative concerns were reported only by a minority of respondents (Fig. 2).

The care provided on the day of surgery was rated predominantly positive by the patients. A total of 40.0% (26 patients) rated the care as “very good,” and another 38.5% (25 patients) as “good.”

**Fig. 3:**
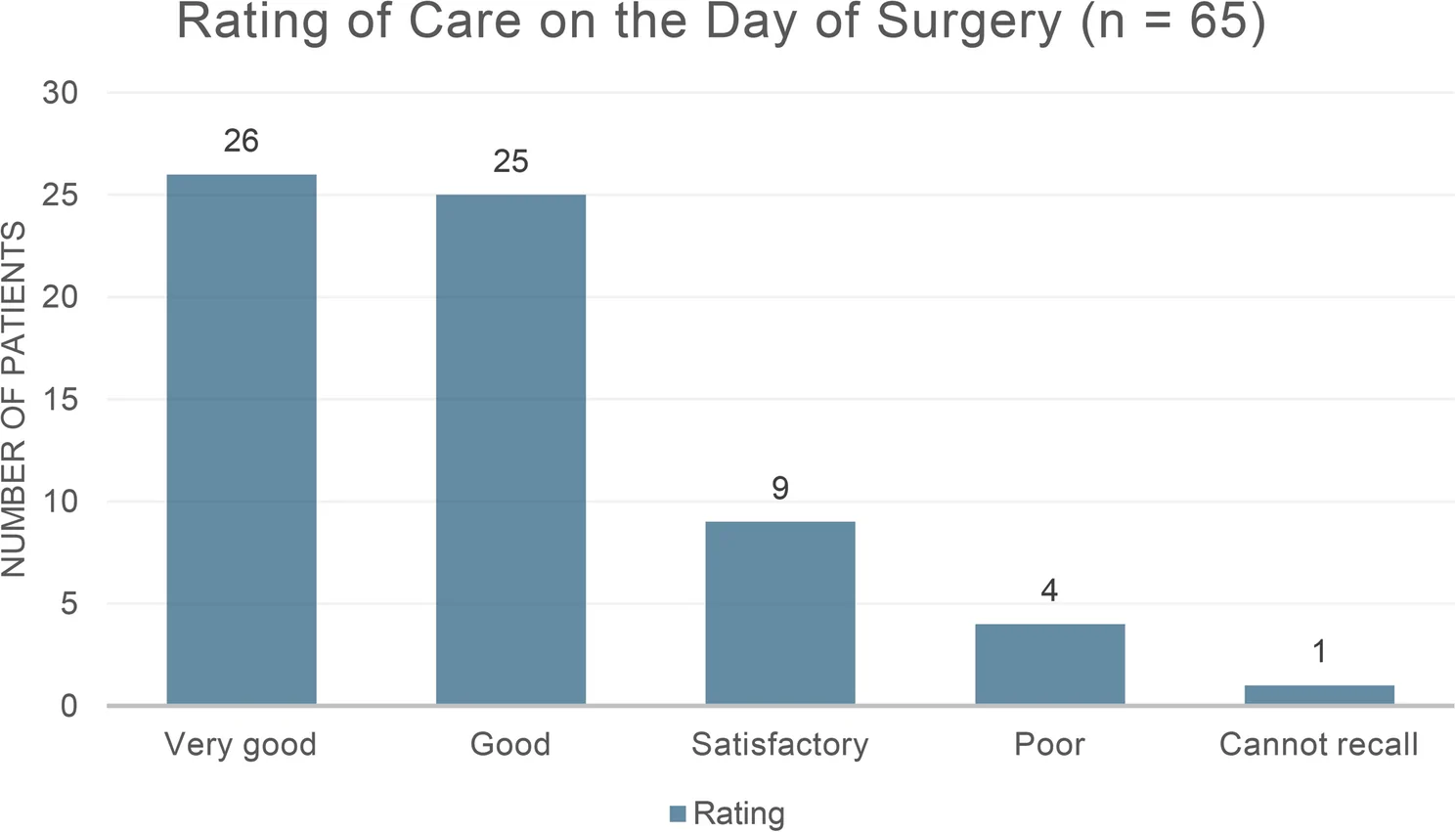
Patient ratings of perioperative care on the day of surgery (*n* = 65)

13.8% (9 patients) considered it “satisfactory,” while only four patients (4/65, 6.2%) rated the care as “poor”. One patient (1.5%) was unable to provide a response (Fig. 3). Overall, most participants expressed satisfaction with the care they received on the day of surgery.

**Fig. 4:**
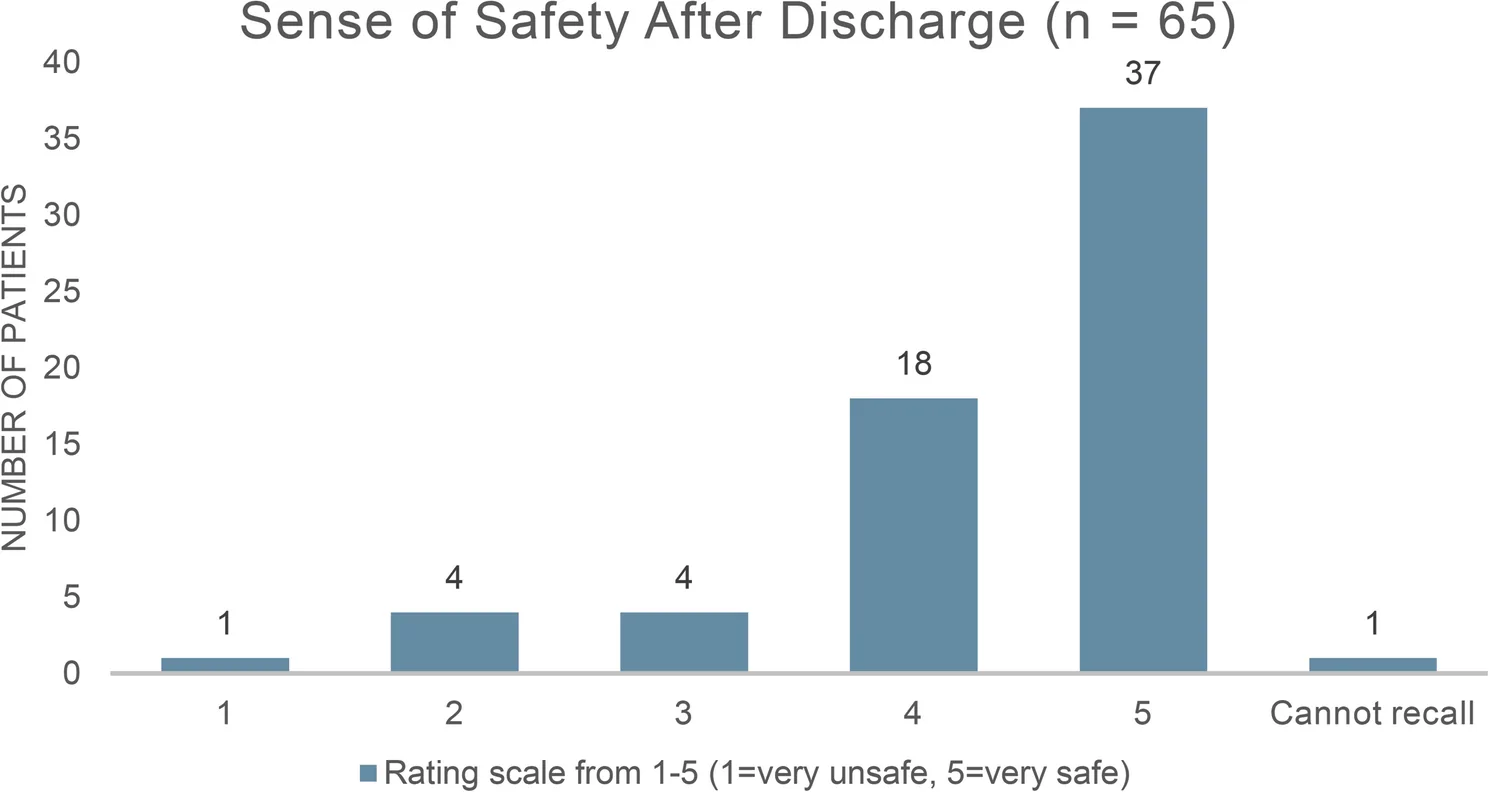
Patient-reported sense of safety after outpatient discharge (*n* = 65)

A central aspect of the survey was the patients’ subjective perception of safety following outpatient discharge. On a scale from 1 (very unsafe) to 5 (very safe), the responses were clearly positive: 84.6% (55 of 65) of patients reported feeling safe or very safe after the procedure (ratings of 4 or 5). Only a small proportion (7.7%, 5 patients) expressed uncertainty (ratings of 1 or 2) (Fig. 4). One patient (1/65, 1.5%) was unable to provide a response.

More than half of the patients (52.3%) reported being fully recovered or fit for work within one to two weeks (34 out of 65 patients). Fifteen patients (23.1%) recovered within four to seven days, and five patients (7.7%) even within three days. In nine cases (13.8%), recovery took longer than two weeks.

**Fig. 5:**
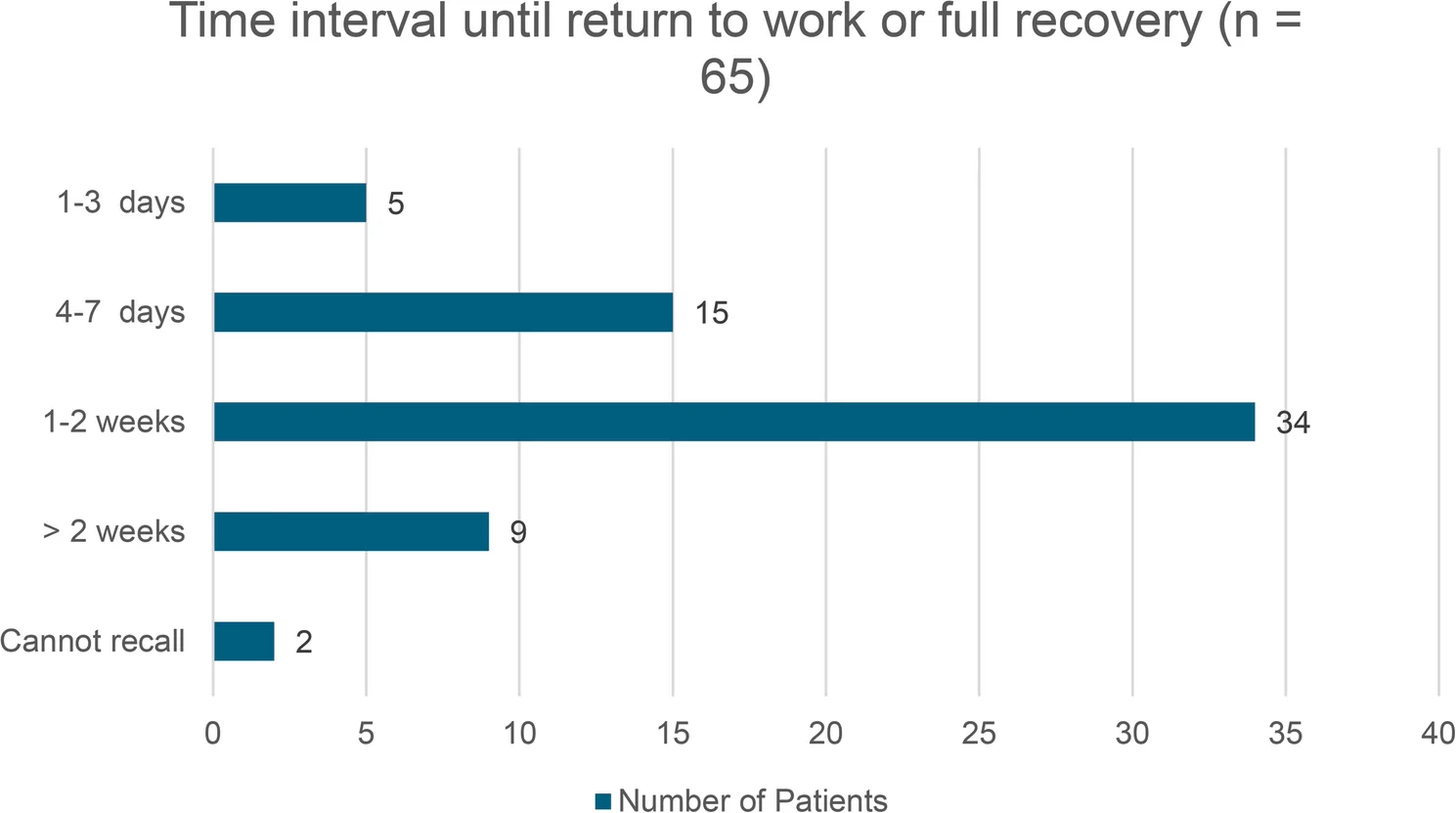
Time to return to work or full recovery after outpatient TEP surgery (*n* = 65)

Two patients (2/65, 3.1%) were unable to provide information on their recovery period (Fig. 5).

**Fig. 6:**
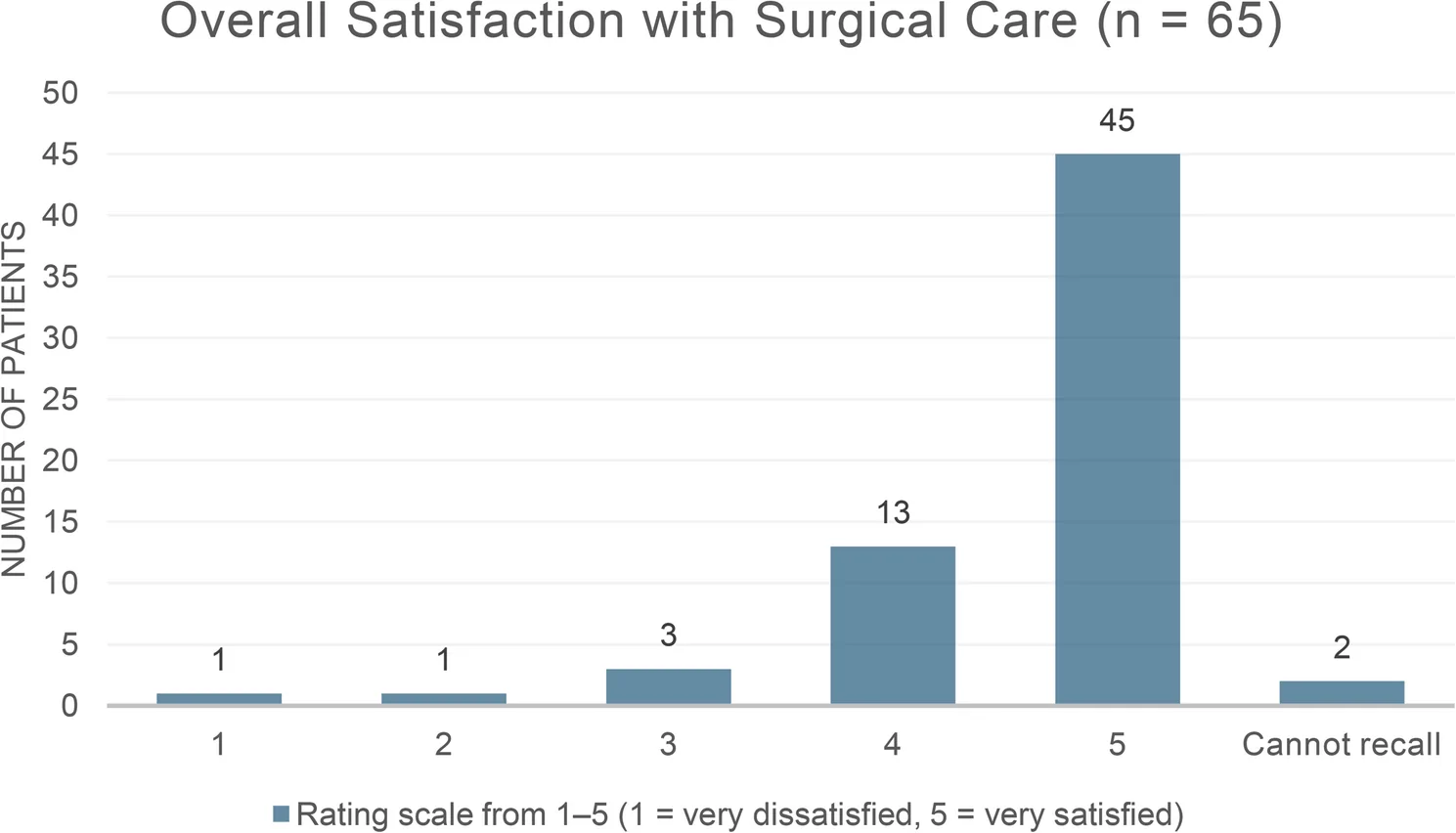
Overall patient satisfaction with outpatient surgical care (*n* = 65)

Overall patient satisfaction among respondents was rated at 4.6 ± 0.8 on a 5-point Likert scale (1 = very dissatisfied, 5 = very satisfied) based on 63 valid responses (Fig. 6). ‘Cannot recall’ responses were excluded from mean score calculation.

**Fig. 7:**
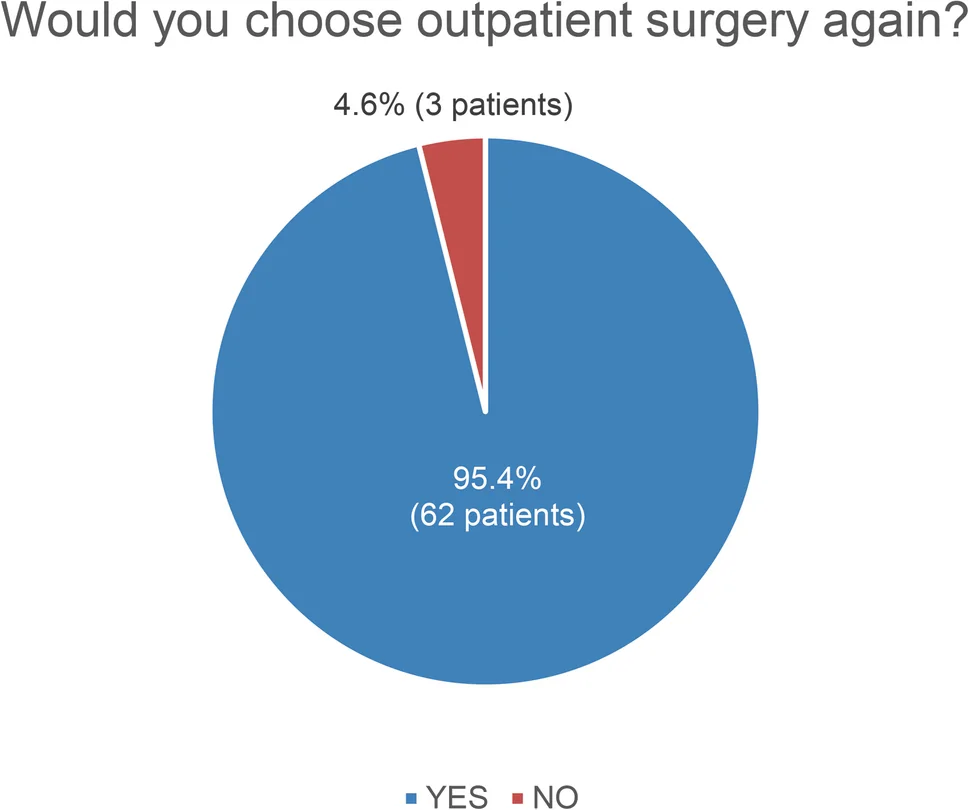
Willingness to undergo outpatient surgery again (*n* = 65)

Most respondents (62/65, 95.4%) stated that they would choose outpatient surgery again (Fig. 7). These patient-reported outcomes should be interpreted in light of the 60.2% response rate and the possibility that respondents were more satisfied or more readily reachable than non-respondents.

### Conversion analysis

The conversion analysis included all 153 planned outpatient TEP procedures performed during the implementation period. Of these, 108 patients were discharged on the day of surgery and constituted the core study cohort, whereas 45 required unplanned inpatient admission, resulting in an overall conversion rate of 29.4%. Conversion rates varied considerably between years; the available data do not establish the reason for this variation, although changes in the threshold for precautionary inpatient monitoring may have contributed. The main reason for admission was suspected intraoperative bleeding requiring observation, followed by technical or anatomical factors, cardiovascular events, and other less frequent causes. No emergency reinterventions were necessary. Details are summarized in Table 4.

**Table 4:** Unplanned conversion from outpatient to inpatient care by year among all planned outpatient TEP procedures (*n* = 153)

Year	Planned outpatient TEPs (*n*)	Conversions (*n*)	Conversion rate (%)
2022	17	4	23.5
2023	75	8	10.7
2024	31	22	71.0
2025	30	11	36.7
**Total**	**153**	**45**	**29.4**

Unplanned conversion from outpatient to inpatient care occurred in 45 of 153 planned procedures (29.4%). The most frequent reason was suspected intraoperative bleeding requiring observation, accounting for 64.4% of all conversions. This category reflected precautionary inpatient monitoring based on an intraoperative concern rather than a confirmed clinically significant hemorrhagic complication. Because the core cohort comprised only patients discharged on the day of surgery, the four intraoperative bleeding events reported in Table 3 and the 29 bleeding-related admissions in Table 5 refer to different patient groups and do not overlap. Technical or anatomical factors were responsible for 13.3%, while cardiovascular events accounted for 8.9%. Other medical reasons, postoperative pain or nausea, and social or organizational factors were rare (Table 5).

**Table 5:** Reasons for unplanned conversion to inpatient care (*n* = 45)

Reason for admission	Patients (*n*)	Percentage (%)
Suspected intraoperative bleeding requiring observation	29	64.4
Technical or anatomical reasons	6	13.3
Cardiovascular events	4	8.9
Other medical reasons	3	6.7
Postoperative pain or nausea	2	4.4
Social or organizational reasons	1	2.2
**Total**	**45**	**100**

## Discussion

This preliminary single-center study provides descriptive data on the early implementation of an outpatient TEP pathway. Among the 108 patients who successfully completed same-day discharge, no life-threatening bleeding, emergency reoperation, or ICU admission occurred; patient-reported outcomes were available for 65 respondents. Published complication rates after TEP vary across studies. Data from the Herniamed Registry reported an intraoperative complication rate of 0.82%, while a prospective series reported no major complications and vascular or organ injury rates of 0.11% [8–10].

Our findings are consistent with current European and international recommendations for groin hernia management, which emphasize evidence-based standardization, careful patient selection, surgeon expertise, and individualized risk assessment when selecting the surgical approach and perioperative setting [6, 7, 11, 12]. Although these guidelines do not specifically address the German AOP reimbursement framework, their emphasis on structured care and individualized risk assessment is consistent with the principles underlying structured outpatient pathways for carefully selected patients undergoing TEP repair. In particular, our exclusion of patients with relevant anticoagulation therapy or coagulation disorders reflects the guideline-based need for caution in patients at increased bleeding risk.

Low postoperative pain scores and relatively short reported recovery times were observed among the evaluated patients; however, the descriptive study design does not allow conclusions regarding the effectiveness of the perioperative pathway. In our cohort, more than half of the patients returned to work within 1–2 weeks. These findings are consistent with previous reports: Jones et al. described a median return-to-work time of 7 days and a mean of 12 days postoperatively [13].

Two early recurrences (1.9%) were observed in our study, which is within the range reported internationally. Recurrence rates following laparoscopic hernia repair in the literature vary between 1% and 4.3% [14].

Earlier studies have suggested that outpatient TEP procedures can be associated with increased levels of anxiety and stress when preoperative preparation is inadequate [15]. Among respondents, perceived safety and overall satisfaction were high. Although structured preoperative counseling and perioperative support formed part of the institutional pathway, the present study was not designed to determine whether these measures contributed causally to the reported patient-reported outcomes. These satisfaction levels are in line with previous literature on patient-reported satisfaction after inguinal hernia repair [16].

An important implication of our findings is that the transition from inpatient to outpatient TEP should not be understood as a purely administrative change. Rather, implementation of an outpatient TEP pathway requires consideration of perioperative processes including patient selection, preoperative counseling, discharge criteria, and postoperative follow-up. The pathway evaluated in this study comprised same-day discharge together with a scheduled postoperative day-1 clinic review and should therefore be interpreted as a combined model of care rather than same-day surgery alone. Standardization of these processes may be relevant for future evaluation, but its impact on safety, admission rates, and patient-reported outcomes cannot be determined from the present dataset. No formal health-economic analysis was performed; therefore, potential cost advantages cannot be concluded from the present data.

Initial concerns regarding the safety of endoscopic hernia repair in the outpatient setting—particularly related to postoperative bleeding—were not accompanied by life-threatening bleeding, emergency reoperation, or ICU admission in the selected core cohort. However, because this was a descriptive study without an inpatient comparator and because conversion rates varied markedly over time, the present data cannot formally exclude an increased risk associated with outpatient TEP.

Analysis of unplanned admissions provides important context for interpreting the outpatient pathway. Overall, 45 of 153 planned outpatient procedures (29.4%) resulted in inpatient admission, a rate that is high for a planned outpatient programme and should temper the interpretation of implementation success. In 29 of 45 admissions (64.4%), the reason was suspected intraoperative bleeding requiring observation, reflecting a low threshold for precautionary inpatient monitoring rather than a confirmed clinically significant hemorrhagic complication. Importantly, no life-threatening bleeding, emergency reoperation, or ICU admission occurred. In the present programme, inpatient admission served as a precautionary fallback when same-day discharge was considered inappropriate. However, the overall admission rate of 29.4%, together with its marked year-to-year variability, indicates that the outpatient pathway was not consistently completed as planned during the study period and limits conclusions regarding successful implementation. The year-to-year variation, including a conversion rate of 71.0% in 2024, cannot be explained definitively from the available retrospective data. Changes in admission thresholds may have contributed, but operational changes were not systematically documented.

The conversion rate observed in our institution contrasts with the large specialized day-surgery series reported by Wakasugi et al., in which 2,148 patients underwent single-incision TEP with tumescent local anesthesia and all were discharged on the day of surgery [17]. Notably, the Wakasugi series included patients with bilateral or recurrent hernias, previous lower abdominal surgery, antithrombotic therapy, and ASA physical status ≥ III, whereas our institutional pathway applied more restrictive eligibility criteria. Differences in institutional setting, surgeon experience, operative technique, perioperative analgesia, and discharge thresholds may contribute to the discrepancy. However, the studies were not designed for direct comparison, and these potential explanations cannot be tested using the present dataset. This study is limited by its retrospective, single-center design, the absence of a comparator group, the moderate response rate, and the potential for responder and recall bias. The interviews were conducted in April and May 2025 after operations performed between January 2022 and May 2025; therefore, recall intervals differed substantially across participants. Because the interviews were conducted by personnel affiliated with the treating department rather than by independent study staff, social-desirability bias cannot be excluded. The characteristics of respondents and non-respondents were not compared, and satisfaction and perceived safety may have been overestimated if more satisfied or more readily reachable patients were more likely to participate. With 65 respondents, the sample size does not permit robust subgroup analyses or inferential comparisons. The study also did not formally compare patients discharged as planned with those requiring unplanned admission, because the available retrospective datasets were not structured for reliable linked subgroup analyses. Although all patients were routinely scheduled for postoperative day-1 review, actual attendance was not systematically documented and a reliable attendance rate cannot be reported. No formal health-economic evaluation was performed. Rare delayed mesh-related complications were not systematically assessed; delayed-onset mesh infection has been reported years after mesh-based hernia repair, underscoring the need for longer-term surveillance [18]. These limitations restrict the generalizability of the findings and require confirmation in prospective multicenter studies. Most importantly, because patient-reported outcomes were obtained only from respondents among patients who successfully completed same-day discharge, these data cannot be used to assess the acceptability of the outpatient programme as a whole.

From a governance perspective, implementation of outpatient TEP in Germany requires more than inclusion of the procedure in the AOP framework. Relevant prerequisites include institutional experience with endoscopic hernia repair, standardized discharge criteria, access to unplanned inpatient monitoring when needed, and reliable postoperative follow-up structures. Therefore, the findings of this single-center study should not be interpreted as sufficient evidence to establish national guidelines on their own. Rather, they provide a real-world implementation experience that may inform future multicenter studies, registry-based evaluations, and national standardization efforts. Future governance strategies should define minimum structural requirements, risk-adapted patient selection criteria, and quality indicators such as unplanned admission, emergency reoperation, readmission, postoperative bleeding, recurrence, postoperative pain, and patient-reported satisfaction.

### Exploratory risk-stratified outpatient TEP pathway

**Fig. 8:**
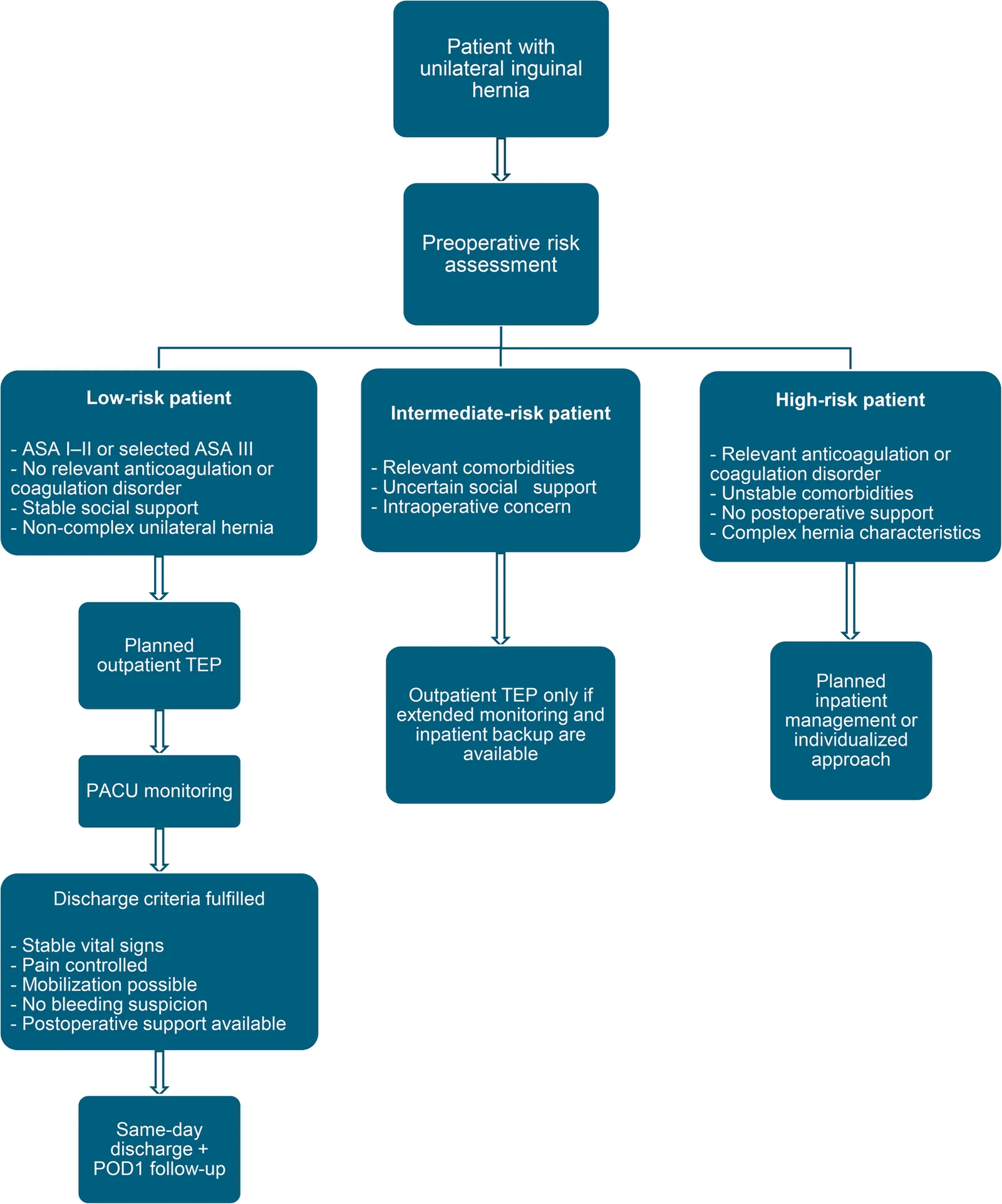
Exploratory risk-stratified pathway for future evaluation of outpatient TEP in Germany

Based on our institutional experience, we outline an exploratory risk-stratified outpatient TEP pathway as a hypothesis-generating framework for future prospective evaluation in Germany. Low-risk patients with unilateral inguinal hernia, ASA I–II or selected ASA III status, no relevant anticoagulation therapy or coagulation disorder, and adequate postoperative social support may be suitable for planned outpatient TEP. Intermediate-risk patients, such as those with relevant comorbidities, uncertain postoperative support, or intraoperative concerns, may undergo outpatient surgery only if extended monitoring and low-threshold inpatient admission are available. High-risk patients, including those with relevant anticoagulation therapy, coagulation disorders, unstable comorbidities, lack of postoperative support, or complex hernia characteristics, should be considered for planned inpatient management or an individualized treatment approach. This exploratory pathway is summarized in Fig. 8 and should not be interpreted as a validated clinical model or national guideline; rather, it is intended solely as a hypothesis-generating framework for future prospective evaluation.

## Conclusion

This preliminary single-center study provides descriptive data on the early implementation of an outpatient TEP pathway following the German AOP reform. Among patients who successfully completed same-day discharge and responded to follow-up, patient-reported satisfaction and perceived safety were high. However, the 29.4% unplanned inpatient admission rate, the exclusion of admitted patients from the patient-reported outcome analysis, the 60.2% response rate, the absence of comparative analyses, and the retrospective single-center design substantially limit conclusions regarding the overall safety, acceptability, and successful implementation of the programme. The proposed risk-adapted pathway should therefore be regarded as exploratory and hypothesis-generating rather than as a validated clinical model. Prospective multicenter studies with predefined admission criteria, systematic follow-up of all patients, and comparative analyses are required before broader recommendations can be made.

## Supplementary Information

Supplementary Material 1.

## Data Availability

The datasets used and/or analyzed during the current study are available from the corresponding author on reasonable request.

## Abbreviations

TEP: Total extraperitoneal repair
PACU: Post-anesthesia care unit
ASA: American Society of Anesthesiologists
NRS: Numeric Rating Scale
POD1: Postoperative day one
