# Supplementary material for: Outpatient Total Extraperitoneal (TEP) for inguinal hernia repair following federal German outpatient operation procedure reform: a preliminary single-center descriptive study of same-day discharge, unplanned admission, and patient-reported outcomes
Source: BMC Surg. 2026 Aug 27;26:580. doi: 10.1186/s12893-026-04161-y (PMC13520304; doi:10.1186/s12893-026-04161-y)
Supplement: Supplementary file 1 [file 12893_2026_4161_MOESM1_ESM.pdf]

Supplementary File 1: Patient Questionnaire (Telephone Interview)

General Information

Patient name: ______________________________

Date of surgery: ____________________________

Type of surgery: outpatient

Preoperative information and counseling

1. How well informed did you feel about the outpatient/inpatient procedure?

☐ Very good ☐ Good ☐ Adequate ☐ Poor ☐ Very poor

2. Did you have concerns before the procedure?

☐ No ☐ Mild concerns ☐ Significant concerns

Postoperative course

3. How would you rate the care on the day of surgery?

☐ Very good ☐ Good ☐ Adequate ☐ Poor ☐ Cannot recall

4. How safe did you feel after discharge at home?

Scale 1 (very unsafe) – 5 (very safe): ___ ☐ Cannot recall

5. Were there any complications or unplanned medical visits?

☐ Yes ☐ No

6. When were you able to return to work?

☐ 1–3 days ☐ 4–7 days ☐ 1–2 weeks ☐ >2 weeks ☐ Cannot recall

Satisfaction and acceptance

7. Overall, how satisfied were you with the surgical care?

Scale 1 (very dissatisfied) – 5 (very satisfied): ___ ☐ Cannot recall

8. Would you choose this surgical approach again?

☐ Yes ☐ No

9. What was the greatest advantage for you?

☐ Early discharge ☐ Less hospital environment

10. What was the greatest disadvantage for you?

☐ Uncertainty ☐ Less monitoring ☐ Organizational issues (family, pets, etc.)
